# Dydrogesterone versus micronized vaginal progesterone for luteal phase support in artificial cycle frozen embryo transfer (REMODEL): a pilot prospective randomized controlled trial

**DOI:** 10.3389/fendo.2026.1785932

**Published:** 2026-04-16

**Authors:** Caroline Roelens, Shari Mackens, Panagiotis Drakopoulos, Lisbet Van Landuyt, Michel De Vos, Herman Tournaye, Christophe Blockeel

**Affiliations:** 1 Brussels IVF, UZ Brussel, Brussels, Belgium; 2 Research Group Genetics Reproduction and Development (GRAD), Vrije Universiteit Brussel, Brussels, Belgium; 3 Institute of life, IVF unit, Athens, Greece; 4 Faculty of medicine, European University Cyprus, Nicosia, Cyprus

**Keywords:** artificial cycle, frozen embryo transfer, luteal phase, micronized vaginal progesterone, oral dydrogesterone

## Abstract

**Background:**

The introduction of vitrification has markedly increased frozen embryo transfer (FET) cycles, driving efforts to optimize FET protocols. In artificial-cycle FET (AC-FET), micronized vaginal progesterone (MVP) is widely used for luteal phase support (LPS), though local side effects are common. Dydrogesterone (DYD), an oral selective progesterone receptor agonist, offers patient-friendly administration, but its efficacy in AC-FET remains uncertain.

**Methods:**

In this single-centre trial (October 2021 - September 2023), women <41 years with normal BMI undergoing single blastocyst AC-FET were randomized (1:1) to DYD 10 mg three times daily (Group A) or MVP 200 mg twice daily (Group B). All received estradiol valerate for endometrial preparation. The primary outcome was ongoing pregnancy rate (OPR) at 12 weeks.

**Results:**

Of 167 screened, 150 were randomized (Group A: 73; Group B: 77). Baseline and cycle characteristics were comparable. Four women switched LPS post-randomization; both per-protocol and intention-to-treat analyses were performed. OPR was 31·5% with DYD vs 45·2% with MVP (p=0·09; difference −13%, 95% CI −38 to 12). ITT analysis was consistent (31·1% vs 44·7%).

**Conclusion:**

Although not statistically significant, the results of this pilot prospective randomized controlled trial may have clinical implications and highlight the need for larger studies investigating the ideal dose and administration route of different LPS medications in AC-FET cycles. Given the differences in pharmacological profiles, varying dosages and more frequent administration of DYD may also warrant exploration.

## 1 Introduction

Frozen embryo transfer (FET) cycles have increased significantly in recent years (1), mainly due to improved cryopreservation techniques and a rise in indications for performing a freeze-only strategy, such as preimplantation genetic testing (PGT), avoidance of ovarian hyperstimulation syndrome (OHSS) (2), and elevated late follicular progesterone levels (3). As a result, the optimization of FET cycles has become an important research area within assisted reproductive technology (ART).

Endometrial preparation for embryo implantation in FET cycles can be achieved through either a natural cycle (NC) or an artificial cycle (AC) (4). Recent studies have demonstrated that artificial cycles are associated with a higher incidence of obstetric complications, including macrosomia, postpartum hemorrhage, and hypertensive disorders of pregnancy, compared to natural cycles (5). This increase in risk is likely attributable to the absence of a corpus luteum, which secretes essential vasoactive substances critical for cardiovascular and renal adaptation during the pre-implantation phase and early pregnancy (6). Consequently, many fertility centers now prefer natural cycles for endometrial preparation prior to FET, particularly in normo-ovulatory patients (7). However, natural cycles are not feasible for patients who are menopausal, have premature ovarian insufficiency (POI), or other underlying conditions that compromise follicular growth. Therefore, continued research into optimizing artificial cycles for such patients remains essential, especially given their increased risk of obstetrical complications, often due to the use of donor eggs or infertility-related factors (8).

Luteal phase support (LPS) in AC-FET cycles is crucial and relies entirely on exogenously supplemented progestins, as follicular growth is mostly suppressed due to the administration of estradiol. Globally, vaginal progesterone remains the most commonly used route for LPS in ART, followed by intramuscular and subcutaneous progesterone. Oral progestin formulations, in contrast, have historically seen limited use, mainly due to the low bioavailability (<5%) of micronized progesterone after hepatic first-pass metabolism (9). However, dydrogesterone (DYD), a retro-progesterone and selective progesterone receptor agonist, offers significantly higher oral bioavailability due to its unique structure, making it a suitable candidate for LPS (10). Two large randomized controlled trials (RCTs) have demonstrated the non-inferiority of DYD compared to MVP in ART cycles with fresh embryo transfer (10, 11). Nevertheless, data regarding the effectiveness of DYD in AC-FET cycles are limited and inconsistent, with varying LPS dosages complicating the interpretation of results (12–17).

Progesterone levels during the mid-luteal phase have been widely researched regarding the success of AC-FET cycles. Low serum progesterone, often defined as below 10 ng/mL, has been associated with lower ongoing pregnancy rates and higher miscarriage rates (18). When MVP is used, serum levels can fluctuate widely among patients and vary during the day (19). Factors such as body mass index (BMI), parity, ethnicity, and smoking status may influence the absorption of MVP (20), complicating personalized LPS protocols. Despite these variations, progesterone levels following MVP administration appear stable across AC-FET cycles within individual patients, suggesting that repeated measurements may not be necessary (21).

Conversely, DYD levels cannot be assessed with standard immunoassays, requiring the use of specialized methods. Studies using these assays have examined the pharmacokinetics of oral DYD, revealing rapid absorption and clearance of both DYD and its active metabolite, 20α-dihydrodydrogesterone, in a consistent 1:25 ratio (22, 23). Similar to vaginal progesterone, interindividual variation in DYD absorption exists, but this variation does not correlate with BMI or body weight. Recent data suggest that lower DYD/DHD levels are associated with poorer pregnancy outcomes, with 24·7% of patients exhibiting low serum DYD/DHD levels (22). Whether the latter can be overcome by administration of a higher or more frequent dose deserves further investigation.

Given the limited available evidence to inform an accurate power calculation for DYD use in AC-FET cycles, this study was designed as a pilot randomized controlled trial to inform the design of future adequately powered studies. This study will compare the efficacy of DYD versus MVP as LPS in AC-FET cycles, with ongoing pregnancy rates as the primary endpoint. Additionally, progesterone levels will be measured at multiple time points for both MVP and DYD to better understand their impact on pregnancy success.

## 2 Materials and methods

### 2.1 Procedures

#### 2.1.1 Study design and setting

The study was conducted at a university-affiliated fertility center and was designed as a pilot prospective randomized controlled trial, aiming to include 150 patients. The first patient was randomized in October, 2021, and recruitment was completed in June, 2023. Informed consent was obtained from all participants prior to their inclusion in the study, and ethical approval was granted by the local ethics committee of the University Hospital of Brussels in March 2021.

#### 2.1.2 Study population, screening and enrollment

**Figure 1:**
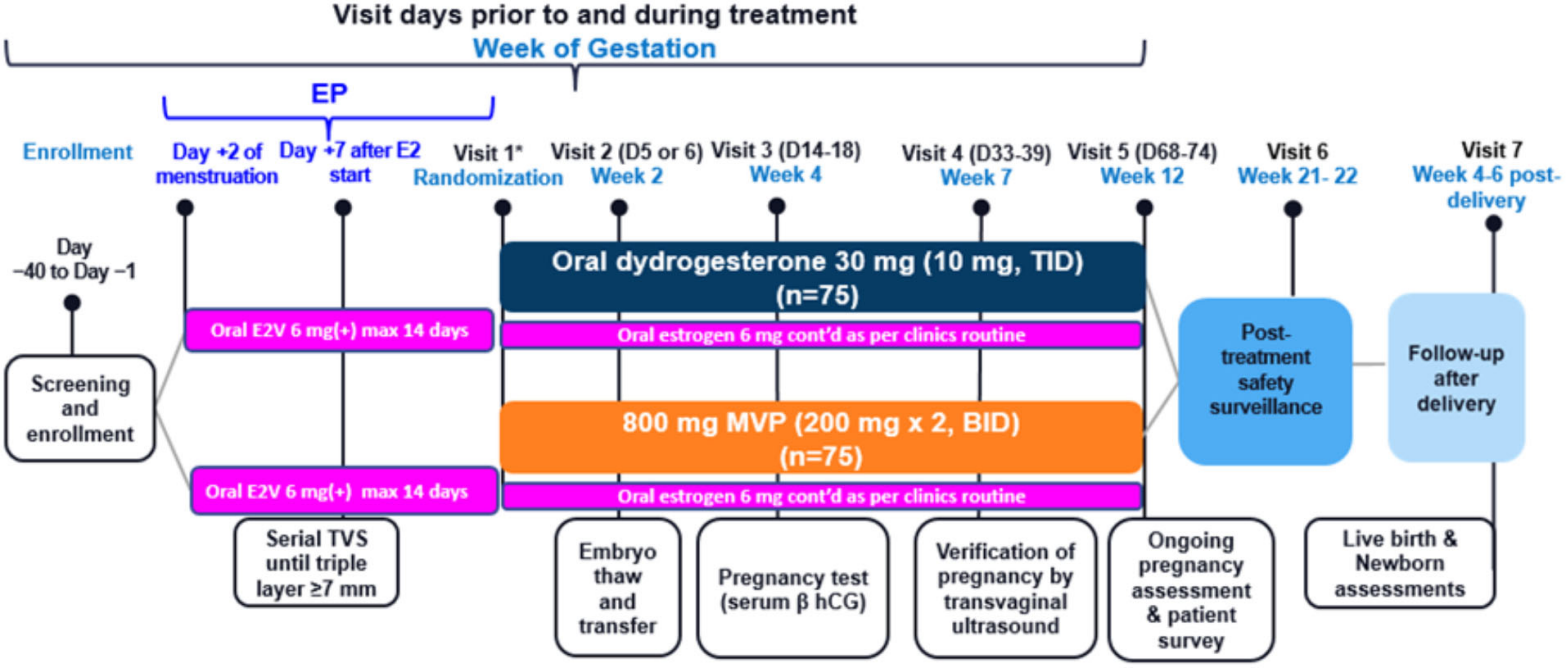
Remodel study schedule. *LPS is administered on the day after randomization BID, twice daily; EP, estrogen priming; E2, estrogen; E2V, estradiol valerate; hCG, human chorionic gonadotropin; MVP, micronized vaginal progesterone; TBC, to be confirmed; TID, three times daily; TVS, transvaginal sonography.

The study population included women undergoing a FET cycle in an artificially prepared cycle (AC). All eligible women were informed about the study by the fertility doctor at the outpatient clinic or at the start of the frozen cycle. Prior to participating in the study and undergoing endometrial preparation recent normal blood investigations were required, including prolactin (PRL), testosterone (T), thyroid-stimulating hormone (TSH), and anti-Müllerian hormone (AMH), with results no older than 12 months. Eligible participants were women aged ≤40 years at the time of IVF/ICSI treatment, with a BMI between 18 and 30 kg/m² and a documented history of infertility. Patients must have undergone controlled ovarian stimulation (COS) as part of ART and either experienced an unsuccessful fresh embryo transfer in that cycle or undergone a freeze-all strategy. They were required to be scheduled for frozen embryo transfer (FET) with a standard exogenous/programmed hormonal replacement therapy (HRT) regimen and to have at least one blastocyst vitrified on day 5 or day 6 after oocyte retrieval. Only elective single blastocyst transfers (SET) were included. A normal ultrasound examination at enrollment (or performed within the last 12 months) was mandatory. Finally, patients had to provide signed consent for the use and disclosure of their data. Women were excluded if they had a history of recurrent miscarriage, defined as more than two consecutive pregnancy losses (biochemical pregnancies not included), or if they had experienced absence of implantation (negative serum β-hCG) after two consecutive IVF/ICSI, or FET cycles in which more than four cleavage-stage embryos or more than two blastocysts had been cumulatively transferred. Patients with untreated hydrosalpinx, endometrial abnormalities detected during ovarian stimulation (such as polyps, submucosal fibroids, hyperplasia, fluid accumulation, or adhesions), or contraindications to pregnancy were excluded. Additional exclusion criteria included participation in another clinical study, known hypersensitivity to DYD or other progestogens, or any contraindication to DYD use as described in the local product label. Women with mental disability or deemed unfit by the investigator to participate, those with a history of prior chemotherapy, and those scheduled for transfer of more than one embryo were also excluded. Patients were randomized to either the MVP group or the dydrogesterone group after eligibility was confirmed and informed consent was obtained. The randomization sequence and allocation were generated using a sealed envelope system with a 1:1 allocation ratio (**Figure 1**). Access to the sealed envelope system was restricted exclusively to the study nurses. Neither the treating physicians nor the patients had access to the envelopes or the order of randomization. Following randomization, patients were not blinded to their treatment group. All patients undergoing frozen embryo transfer (FET) provided informed consent for the collection and storage of blood samples throughout their treatment.

#### 2.1.3 Study objectives

The primary endpoint was ongoing pregnancy, defined as the presence of fetal cardiac activity on pelvic (vaginal or abdominal) ultrasound at 12 weeks of gestation. Secondary endpoints included live birth rate (delivery of a live infant after 22 weeks), biochemical pregnancy or positive hCG rate (serum β-hCG >25 mIU/mL without ultrasound confirmation), biochemical pregnancy loss, clinical pregnancy (≥1 gestational sac on transvaginal ultrasound; specified in the protocol but not included in the final analysis), miscarriage (spontaneous loss of a clinical pregnancy before 12 weeks, excluding biochemical losses), obstetric complications (hypertensive disorders of pregnancy), tolerability and safety, and congenital malformations. In addition, we investigated the association between plasma concentrations of dydrogesterone and 20α-dihydrodydrogesterone on the day of embryo transfer and subsequent pregnancy outcomes.

#### 2.1.4 Ovarian stimulation and embryo vitrification

Embryos were obtained following ovarian stimulation using either a GnRH agonist or antagonist protocol, combined with gonadotrophins (recombinant FSH or highly purified menopausal gonadotrophins, HPhMG). After oocyte retrieval, fertilization was performed via *in vitro* fertilization (IVF) or intracytoplasmic sperm injection (ICSI), with the embryos cultured to the blastocyst stage (day 5 or 6). A fresh embryo transfer was conducted, or in cases where the patient had a high risk of ovarian hyperstimulation syndrome (OHSS) or other medical condition (i.e elevated serum progesterone levels at the time of ovulation triggering), a freeze-only strategy was utilized.

Cryopreservation of surplus blastocysts was performed using closed vitrification, as described by Van Landuyt et al. (24). This was done only for blastocysts that reached full development, with a well-defined inner cell mass (ICM) and trophectoderm (TE), classified as BI3BB under the Gardner scoring system (25).

On the day of embryo transfer, blastocysts were warmed in the morning, and the transfer was carried out only if at least 50% of the blastocyst’s cells survived the thawing process. Blastocysts were assigned a morphological grade at the time of transfer: top-quality blastocysts were graded AA, AB, or BA based on ICM and TE scores; good-quality blastocysts were graded BB or CA; while poor-quality blastocysts were graded AC, CB, BC, or were not fully re-expanded at the time of transfer. Only patients having at least one top-quality or good quality blastocyst vitrified were included in this study.

#### 2.1.5 Endometrial preparation for frozen embryo transfer

Endometrial preparation was achieved in an AC-FET cycle with administration of oral estradiol valerate (2 mg, three times daily) from early in the menstrual cycle (no later than day 3). Progestin supplementation, aimed at inducing secretory changes in the endometrium and opening the window of implantation, was initiated once the endometrium reached a thickness of at least 7 mm. Prior to the start of progestin treatment, patients were randomized into two groups: Group A received luteal phase support (LPS) with DYD (one tablet of Duphaston^®^ 10 mg, three times daily and 8h interval, for a total daily dose of 30 mg), while Group B received MVP (two capsules of Utrogestan^®^ 200 mg, twice daily and a 12h interval) (**Figure 1**).

Blastocyst transfer was performed on the sixth day of progestin supplementation, and a pregnancy test was conducted 12 days post-transfer. If pregnancy was achieved, LPS continued until 12 weeks of gestation. Ultrasonographic evaluations were carried out at 7 weeks to confirm viable intrauterine pregnancy and at 12 weeks to assess ongoing pregnancy.

#### 2.1.6 Blood sampling during endometrial preparation

Blood samples were collected at Visit 1 (the day of FET, before the onset of luteal phase support) and subsequently at Visits 2-5, as shown in **Figure 1**.

For each collection, 6 ml of blood was drawn into EDTA tubes and immediately handled in an ice bath. Plasma was separated by centrifugation (within one hour of collection) at 4000 rpm for 10 minutes. The plasma was then aliquoted into two samples and stored at -20 °C for later analysis of estradiol (E2), DYD, DHD, and progesterone (P4) using a validated LC-MS/MS method. The samples were then sent to the laboratory NUVISAN (NUVISAN GmbH, Neu-Ulm, Germany) for analysis.

Blood was also collected at Visit 1 or within 12 months prior to this visit to determine routine laboratory values, including hematology (hemoglobin, hematocrit, RBC count, WBC count, platelet count), biochemistry (glucose, creatinine, ALAT, ASAT, uric acid), and endocrinology (PRL, testosterone, TSH, AMH). Hormonal measurements, including FSH, LH, estradiol (E2), and P4, were also determined. Relevant hormone levels (E2, DYD, DHD, and P4) were assessed on Visits 2-5, with blood collected within 2 hours after LPS intake. All sample shipments were sent on dry ice by courier in separate batches directly to NUVISAN for storage and later analysis.

#### 2.1.7 Treatment satisfaction questionnaire for medication

To evaluate the effectiveness, convenience, and overall satisfaction with the LPS medication, patients will complete a questionnaire during Visit 5. The parameters assessed will align with those included in the validated shortened version of the Treatment Satisfaction Questionnaire for Medication (TSQM-9), addressing patient satisfaction in the following areas:

- The ability of the medication to prevent or treat their condition.
- The extent to which the medication relieved symptoms.
- The time taken for the medication to take effect.
- The ease of use of the medication.
- Challenges in planning the timing of medication use.
- The convenience of adhering to the prescribed instructions.
- Confidence in the medication’s therapeutic benefits.
- The perception that the medication’s positive aspects outweigh any negative aspects.
- Overall satisfaction with the medication.

The scores for effectiveness, convenience, and global satisfaction were calculated using the following formulas:

- Effectiveness: [((Question 1 + Question 2 + Question 3) − 3)/18] × 100.
- Convenience: [((Question 4 + Question 5 + Question 6) − 3)/18] × 100.
- Global Satisfaction: [((Question 7 + Question 8 + Question 9) − 3)/14] × 100.

The global satisfaction score specifically reflects the patient’s overall contentment with their treatment, considering all aspects of their experience. This score helps healthcare providers and researchers understand the patient’s perspective on their medication, which can be crucial for improving treatment adherence and outcomes.

##### 2.1.7.1 Assessment of adverse events

Adverse events (AEs) were recorded in REDCap using medical terminology. Events could be identified through participant reports, concomitant medication use, physical examination, or study-specific tests. AEs were collected from the time of informed consent until the last study visit.

Each AE was evaluated for duration, severity, seriousness, and causal relationship to the study drug, as well as action taken, concomitant treatment, outcome, and study discontinuation. Severity was classified as: mild (no interference with daily activities), moderate (some interference), or severe (marked disruption of daily activities).

### 2.2 Statistical analysis and sample size calculation

Studies comparing MVP 800mg/day and DYD 30mg/day using single blastocyst transfer in AC-FET cycles were absent at the time of the initiation of the study. In view of this, an a-priori sample-size calculation was not performed and the current study was designed as a pilot randomized study including 150 patients. Patients were randomized prior to the start of progesterone supplementation and only when endometrial thickness was sufficient (>7.0 mm) after maximal 14days of estradiol priming, to either group A using MVP, or to group B using DYD only after patient eligibility was established and patient consent was obtained. Randomization sequence and allocation was created using a computer generated randomization list in combination with a sealed envelope system, using 1:1 allocation. Analyses were performed in intent-to-treat fashion and per-protocol. Consequently, in the intention-to-treat analysis, all patients will be included in the final analysis as long as after fulfilment of the inclusion criteria they were randomly allocated to one of the treatment groups, whereas the per-protocol analysis will include only those patients who completed the treatment originally allocated.

Descriptive summary measures as mean (standard deviation) were used for continuous variables and number (percentage) for categorical variables, in order to provide a summary estimate of patient demographics and baseline characteristics, in line with the CONSORT statement (www.consort-statement.org). For the primary outcome (ongoing pregnancy rates), the secondary outcomes and the other efficacy endpoints, results were analyzed by using a two-sided chi-square test or Fisher exact test as appropriate, with a level of significance 0.05. For continuous variables, parametric (independent t-test) or non-parametric (Mann–Whitney test) tests were used depending on the normality of the distribution. Normality was examined by the use of the Shapiro–Wilk test.

Multivariable regression analysis was performed for the primary outcome accounting for confounding factors as age, BMI, endometrial thickness and previous delivery.

All analyses were performed in STATA 13 Statistical software.

## 3 Results

### 3.1 Study population and ART treatment characteristics

**Figure 2:**
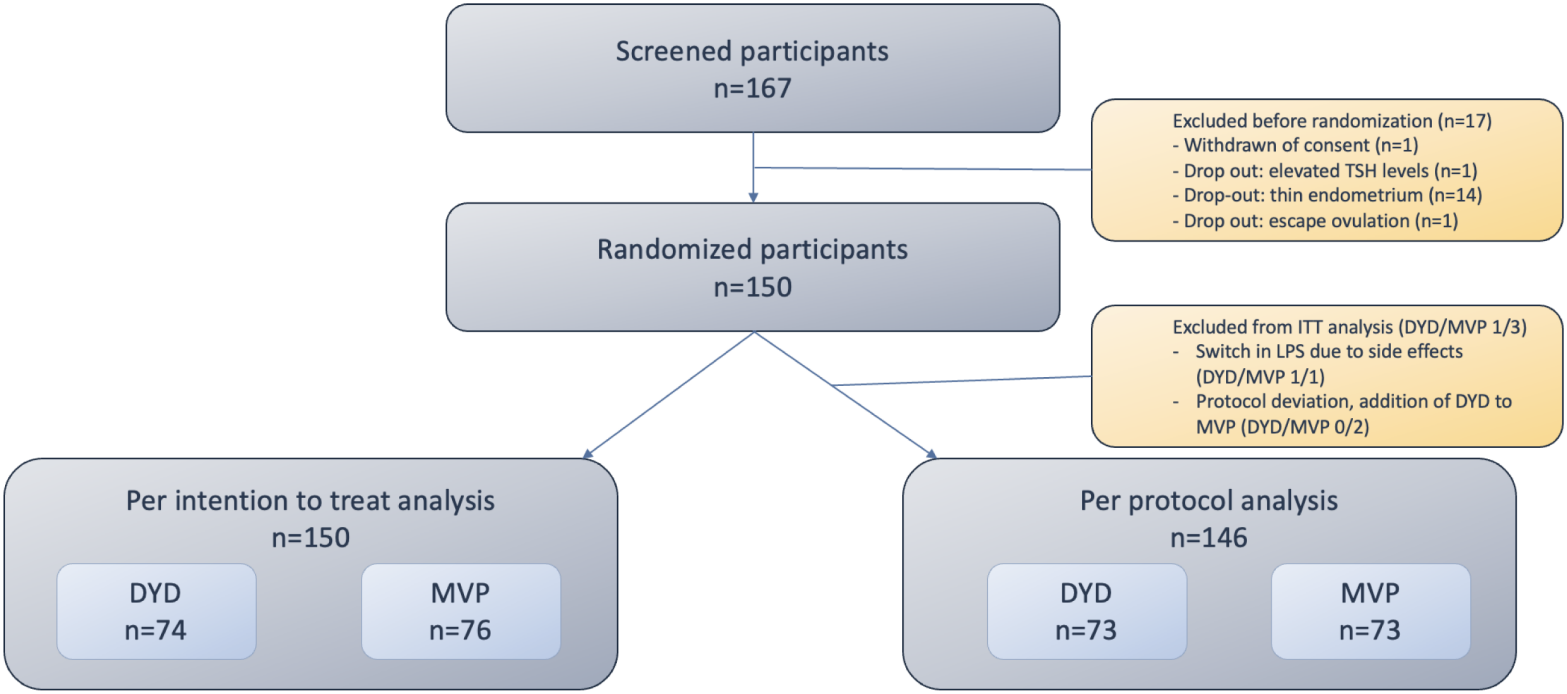
Flowchart of screened and randomized participants. *Excluded from ITT (n=4) DYD group (n=1). Extra MVP from first day pf P test since low P (n=1) MVP group (n=3): Extra DYD started from day of pregnancy test since low P level. Extra DYD from day of FET since low P value. Stop utro since allergic reaction 2 days before FET and switch to DYD.

A total of 167 patients were screened, of which 17 were excluded from randomization: 1 withdrew consent, 1 was excluded due to elevated TSH levels, 14 did not proceed because of insufficient endometrial thickness after 14 days of estradiol priming, and 1 was excluded due to premature ovulation. Of the 150 patients eligible for randomization, 74 were analyzed in group A (DYD group) and 76 in group B (MVP group). In the per-protocol analysis, 146 patients were included, while 4 were excluded: 2 due to a switch in LPS caused by side effects, and 2 due to protocol deviations involving the addition of DYD to MVP during the study period (see **Figure 2** flowchart).

The demographic characteristics of the patients are listed in **Table 1** and were comparable between the two groups due to randomization. The mean age was 31·7 years in group A and 32·4 years in group B.

**Table 1:** Demographic patient and ART treatment characteristics compared for both study groups.

Characteristic	Group A: dydrogesterone (n=74)	Group B: MVP (n=76)	P-value
Age at screening	31·7±3·9	32·4±4·2	
BMI	24·3±3·3	23·6±2·9	
Smoking	4·2%	9·6%	0·33
Previous live birth	26·0%	23·3%	0·70
Type of infertility - Primary infertility - Secondary infertility	52·1% 47·9%	57·5% 42·5%	0·51
Cause of infertility 1. Ovulation dysfunction 2. Tubal 3. Endometriosis 4. Andrological 5. Idiopathic 6. Other	13·7% 6·9% 2·7% 31·5% 37·0% 8·2%	16·4% 5·5% 0% 28·8% 38·4% 11·0%	0·84
Presence of regular cycle	72·6%	76·7%	0·57
Fresh Cycle characteristics			
Number of oocytes retrieved	19·4±9·9	17·6±7·9	0·36
Fertilization method 1. IVF 2. ICSI 3. IVF versus ICSI	5·5% 63·0% 31·5%	6·9% 67·1% 26·0%	0·79
Frozen cycle characteristics			
Rank of FET 1^st^ 2^nd^ 3^th^	84·9% 13·7% 1·4%	87·7% 9·6% 2·7%	0·70
E2 at start (ng/L)	46·2±24·9	40·6±18·1	0·16
P level at start (µg/L)	0·4±0·4	0·4±0·3	0·11
Endometrial thickness at planning (mm)	8·5±1·4	8·3±1·3	0·32
E2 at FET (visit2) (ng/L)	278·7±572·4	206·0±74·5	0·66
P at FET (visit2) (µg/L)	0·3±0·6	13·0±7·1	<0·001
Dydrogesterone level at FET (visit2) (ng/ml)	2·0±1·30	NA	
20a-dyhydrodydrogesterone level at FET (visit2) (ng/ml)	55·00 ±31·63	NA	
E2 at pregnancy test (visit3) (ng/L)	327·9±997·4	195·5±78·6	0·04
P at pregnancy test (visit3) (µg/L)	0·4±0·7	13·8±5·1	<0·001
Dydrogesterone level at pregnancy test (visit3) (ng/ml)	2·1±1·2	NA	
20a-dyhydrodydrogesterone level at pregnancy test (visit3) (ng/ml)	64·9±33·8	NA	
Total number of visits to the clinic	2·3±0·5	2·3±0·7	0·94

Fresh cycle characteristics (**Table 1**) were similar between the groups, with a mean oocyte yield at oocyte pick-up (OPU) of 19·4 ± 9·9 for group A and 17·6 ± 7·9 for group B. Intracytoplasmic sperm injection (ICSI) was the fertilization method used in the majority of participants, accounting for 63·0% in group A and 67·1% in group B.

Frozen cycle characteristics (**Table 1**) showed that 84·9% of patients in group A and 87·7% in group B underwent first-rank blastocyst transfer, with comparable baseline hormonal profiles. A significant difference in progesterone levels was observed at both the time of FET and the pregnancy test between the groups, as DYD levels are not measurable using standard progesterone assays.

Mean progesterone levels in group B at the day of FET were 13·0±7·1µg/L and 13·8±5·1 µg/L the day of first pregnancy test. Estradiol levels the day of the first pregnancy test were 327·9 ± 997·4ng/L and 195·6±78·6ng/L for group A and B respectively (p<0·001).

### 3.2 Clinical outcome measures

The primary outcome of the study, ongoing pregnancy at 12 weeks’ gestation, was not significantly different between the two groups, with rates of 31·5% (23/73) and 45·2% (33/73) for Group A and Group B, respectively, in the per protocol analysis. The intention-to-treat analysis showed similarly non-significant results, with ongoing pregnancy rates of 31·1% (23/74) in Group A compared to 44·7% (34/76) in Group B (p = 0·09, difference in proportions: -13%, 95% CI: -38 to 12). Additionally, multivariable regression analysis was performed for the primary outcome accounting for confounding factors as age, BMI, endometrial thickness and previous delivery and showed no association between type of LPS used and ongoing pregnancy rates (aOR 1·88; 95% CI 0·93-3·81, p=0·08).

Positive hCG rates were comparable between the two groups, 58·9% (43/73) in Group A and 61·6% (45/73) in Group B (p = 0·74). Furthermore, clinical miscarriage rates (before 12 weeks’ gestation) were lower in group B compared to group A (24·4% (11/45) vs. 32·6% (14/43), respectively), although this difference did not reach statistical significance (p = 0.40). As only one late miscarriage (beyond 12 weeks of gestation) was observed, this secondary outcome was not presented separately in the results table. The live birth rate (LBR) also failed to show a statistically significant difference between group A and group B, at 28·8% (21/73) and 42·5% (32/73), respectively (p = 0·08). (**Table 2**).

**Table 2:** Clinical outcome measures.

Outcome measure	Group A dydrogesterone	Group B MVP	P-value
Positive hCG	58·9% (43/73)	61·6% (45/73)	0·74
Biochemical Pregnancy loss	13·9% (6/43)	2·2% (1/45)	0·06
clinical miscarriage before 12w	32·6% (14/43)	24·4% (11/45)	0·40
ONGOING pregnancy at 12w (PP)	31·5% (23/73)	45·2% (33/73)	0·09
ONGOING pregnancy at 12w (ITT)	31·1% (23/74)	44·7% (34/76)	0·09
LBR	28·8% (21*/73)	42·5% (32**/73)	0·08

hCG, Human chorionic gonadotrophin; PP, per protocol; ITT, intention to treat; LBR, Live birth rate.

*TOP deletion chromosome 4 (n=1).

TOP schizencephalie (n=1).

**TOP polymalformation(n=1).

Miscarriage 12w3/7 (n=1).

### 3.3 Clinical outcome measures related to dydrogesterone and 20α-dihydrodydrogesterone levels

**Figure 3:**
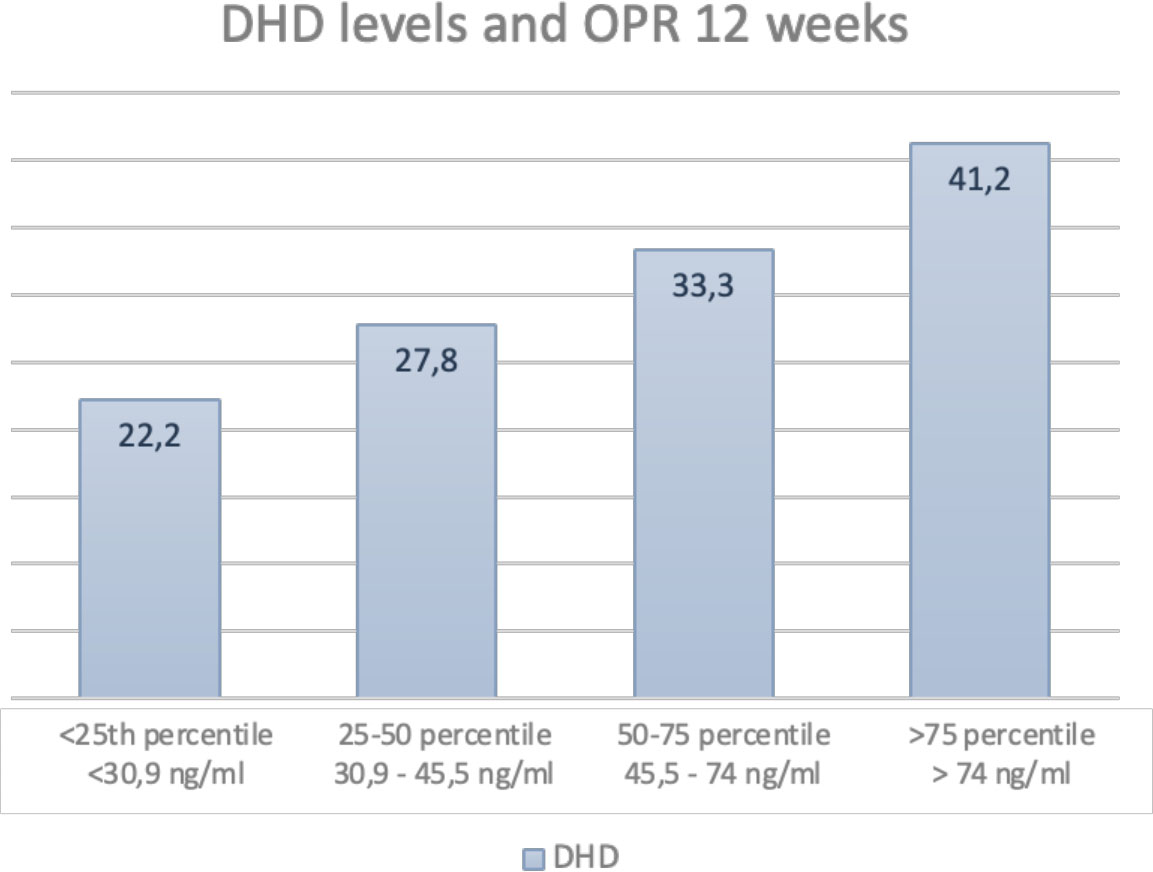
Percentage of OPRs at 12 weeks divided by percentiles of DHD values.

**Figure 4:**
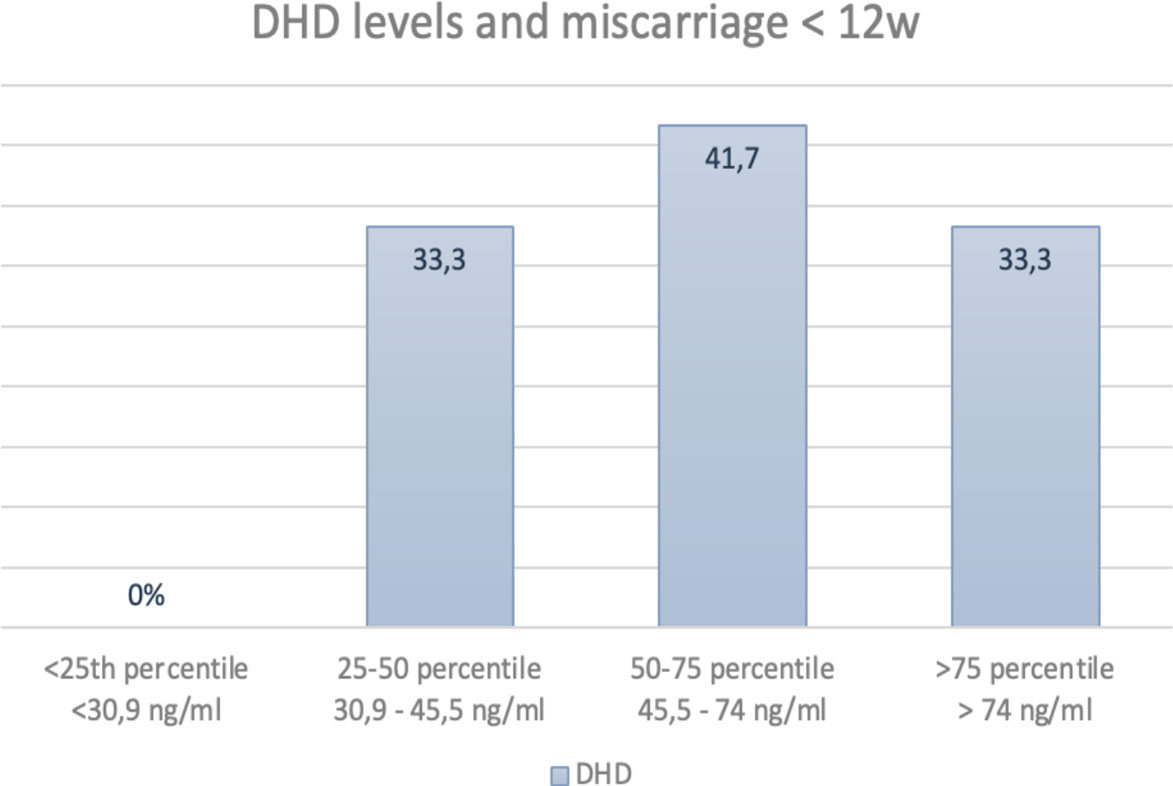
Percentage of clinical miscarriages before 12 weeks divided by percentiles of DHD values.

In order to investigate the influence of different plasma levels of dydrogesterone and 20α-dihydrodydrogesterone measured on the day of embryo transfer on pregnancy outcomes, four quantiles were determined (**Tables 3**, **4**). When focusing on the primary outcome, ongoing pregnancy, a trend toward better outcomes in higher quantiles was observed for both dydrogesterone and 20α-dihydrodydrogesterone. However, due to the low number of patients included in each group, no statistically significant results were obtained. Clinical miscarriages, on the other hand, were not linearly related to the levels of either dydrogesterone or 20α-dihydrodydrogesterone (**Figures 3**, **4**).

**Table 3:** Dydrogesterone levels divided by quantiles and pregnancy outcomes.

Outcome measure	Quantile 1	Quantile 2	Quantile 3	Quantile 4	P-value
Ongoing pregnancy 12w	22·2% (4/18)	25·0% (5/20)	41·2% (7/17)	37·5% (6/16)	0·56
Live birth	22·2% (4/18)	20·0% (4/20)	41·2% (7/17)	31·3% (5/16)	0·50
Clinical miscarriage	0%	27·3% (3/11)	46·2% (6/13)	36·4% (4/11)	0·26

Quantile			Dydrogesterone level		
1 (<25%)			<0·90 ng/ml		
2 (25-50%)			0·90 - 1·69 ng/ml		
3 (50-75%)			1·69 - 2·44 ng/ml		
4 (>75%)			>2·44 ng/ml		

**Table 4:** Dihydrodydrogesterone levels divided by quantiles and pregnancy outcomes.

Outcome measure	Quantile 1	Quantile 2	Quantile 3	Quantile 4	P-value
Ongoing pregnancy 12w	22·2% (4/18)	27·8% (5/18)	33·3% (6/18)	41·2% (7/17)	0·66
Live birth	22·2% (4/18)	22·2% (4/18)	27·8% (5/18)	41·2% (7/17)	0·57
Clinical miscarriage	0%	33·3% (4/12)	41·7% (5/12)	33·3% (4/12)	0·46

Quantile			20α-Dihydrodydrogesterone level		
1 (<25%)			<30·9 ng/ml		
2 (25-50%)			30·9 - 45·5 ng/ml		
3 (50-75%)			45·5 - 74·0 ng/ml		
4 (>75%)			>74·0 ng/ml		

### 3.4 Pregnancy complications

The mean gestational age at delivery was 39·2 weeks in both Group A and Group B. One patient in each group delivered prematurely (<37 weeks). Hypertensive disorders of pregnancy (including pre-eclampsia and gestational hypertension) were more frequent in Group B (12·5% [4/32]) than in Group A (4·8% [1/21]) (p=0.64). First-trimester bleeding was reported by one patient in each group. No cases of gestational diabetes or intrahepatic cholestasis of pregnancy were observed. Ultrasonographic assessments of fetal and neonatal malformations are presented in **Supplementary Table 1**; one neonatal malformation (duodenal malrotation) was diagnosed in Group B.

### 3.5 Safety, convenience and tolerability

Four serious adverse events in three patients were registered in this study. Three occurred in group A (DYD group). The first two involved a twin pregnancy, where one fetus experienced intrauterine fetal demise at 14 weeks of gestation. The second fetus was diagnosed with schizencephaly, which led to a termination of pregnancy at 32 weeks. The third event in the DYD group was an ischemic cerebrovascular accident. The patient presented at six weeks of pregnancy with visual disturbances. An MRI revealed a subacute ischemic lesion in the right occipital region, with minor involvement of the left cortico-frontal region. The patient was treated with acetylsalicylic acid and low molecular weight heparin (LMWH) and was discharged after six days of hospitalization. Estradiol valerate was discontinued due to a suspected estrogenic effect contributing to the event.

In group B (MVP group), one serious adverse event was reported: a child was born with duodenal malrotation, necessitating hospitalization and surgical intervention at six days of life. This was discovered after multiple episodes of vomiting (see **Supplementary Table 1**).

**Figure 5:**
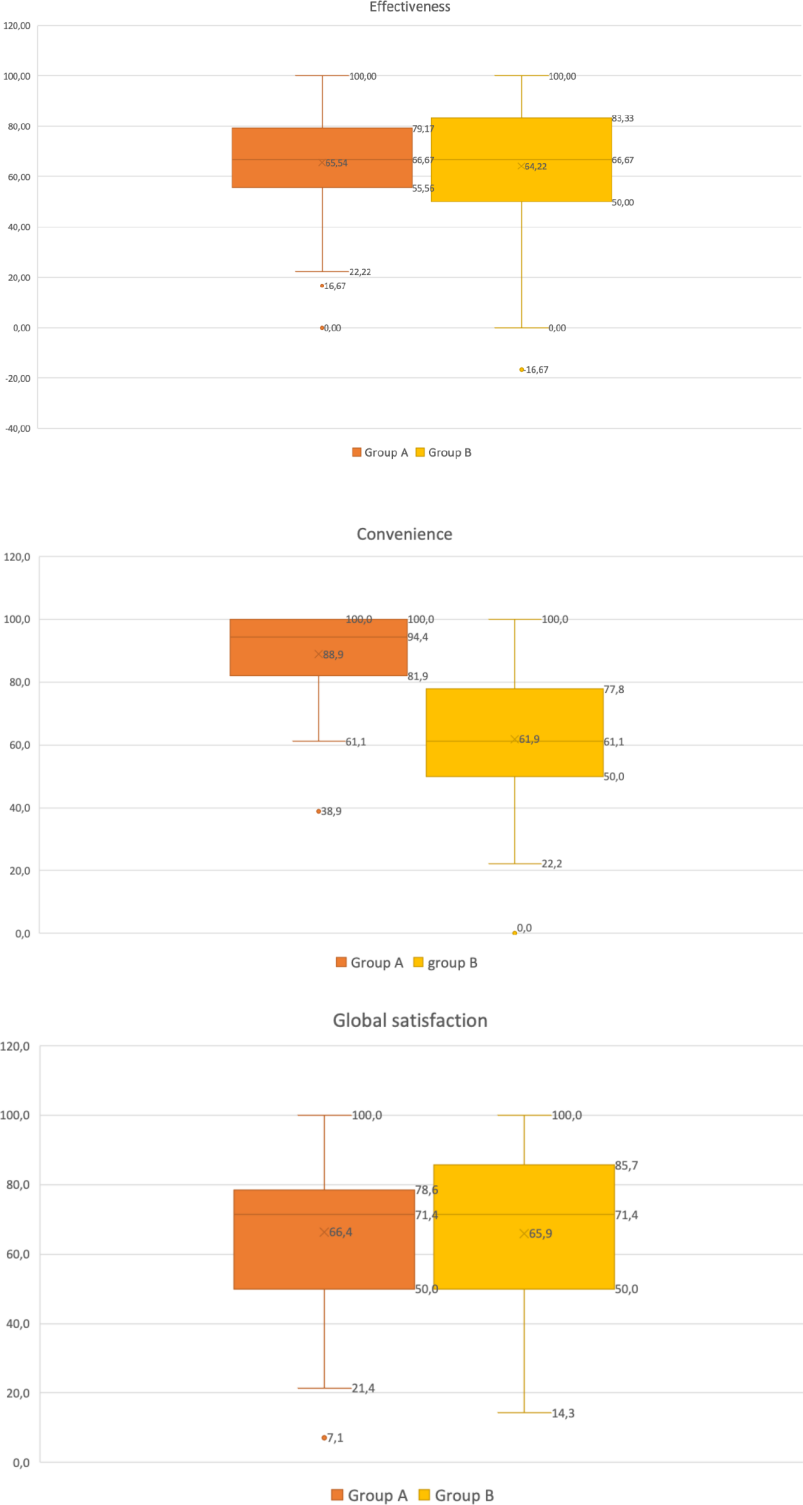
Boxplots comparing effectiveness, convenience, and global satisfaction between Group A (dydrogesterone) and Group B (MVP), based on the Treatment Satisfaction Questionnaire for Medication (TSQM-9).

The effectiveness, convenience, and global satisfaction of both LPS medications were evaluated using the TSQM-9 questionnaire. The mean effectiveness score was 65·5% for Group A and 64·2% for Group B (p=0·73). Convenience was significantly higher in Group A, with a mean score of 88·9%, compared to 61·8% in Group B (p < 0·001). Despite these differences, global satisfaction scores were comparable between the two groups, with 66·4% reported for Group A and 65·9% for Group B (p=0·89) (see **Figure 5**).

## 4 Discussion

Luteal phase support in AC-FET cycles is crucial, yet few studies have investigated the pharmacokinetics and optimal dosing regimens of different progestin formulations for this indication. In this study, DYD at a dose of 30 mg daily was compared with micronized vaginal progesterone (MVP) at a daily dose of 800 mg, a regimen supported by recent publications (26) and generally accepted, though it exceeds the standard registered dose of 200 mg three times daily for luteal phase support in ART. To compensate for the complete absence of endogenous progesterone production in AC-FET cycles, an increase in the MVP dosage was introduced without supportive pharmacokinetic studies. In contrast, dydrogesterone (DYD) dosage was not altered, consistent with evidence from two large randomized controlled trials (RCTs) (10, 11) showing no inferiority of DYD compared to MVP in fresh cycles, as well as the registered dose indication in the manufacturer’s label.

In this pilot randomized controlled trial, no statistically significant difference was observed between DYD and MVP for LPS in AC-FET with respect to positive hCG rates and ongoing pregnancy rates. However, a potentially clinically relevant increase in clinical miscarriage rates was observed in the DYD group, resulting in a trend toward a lower live birth rate. The standard DYD dosing regimen used in this study, extrapolated from evidence in fresh cycles, may not be optimal in artificial cycles, where endogenous progesterone production is absent. Therefore, the observed reduction in live birth rate may, at least in part, be related to insufficient DYD dosing in this specific clinical context. Previously published studies have reported conflicting findings regarding DYD dosing and pregnancy outcomes. Three randomized controlled trials (RCTs) that administered 40 mg of DYD daily found no significant differences in pregnancy outcomes compared to MVP (800mg daily, or MVP gel 90mg twice daily) or intramuscular progesterone (100mg daily) (13, 16, 27). In parallel, retrospective analyses reported similar pregnancy outcomes when 30 mg of DYD was used (15, 17). An older RCT by Zarei et al. (14) involving 400 FET cycles showed lower pregnancy outcomes when 20 mg of DYD was used as the sole LPS agent in AC-FET. These findings suggest that higher DYD dosages than currently indicated may be more effective in AC-FET cycles.

A study by Loreti et al. (19), which investigated the pharmacokinetics of DYD compared to MVP, revealed very rapid absorption (with maximum concentration achieved within 1.5 hours) and rapid clearance of both DYD and its metabolite, DHD, even after 8 days of administration. These fluctuations might suggest the need for more frequent administration of DYD in AC-FET cycles to maintain more consistent plasma levels in order to compensate for the complete absence of endogenous progesterone. Furthermore, Neumann et al. (22) identified a relationship between pregnancy outcomes and plasma levels of both DYD and DHD, with a significant reduction in ongoing pregnancy rates observed in the lowest quartile of plasma levels. However, these observations were not supported by our data, although ongoing pregnancy rates appeared to increase with increasing DYD and DHD levels. No linear relationship could be established for miscarriage rates. The limited number of patients included in this sub-analysis may have affected the results. Whether plasma levels can be increased through more frequent DYD administration than the currently used standard dosing regimen remains unknown, but this question may warrant further investigation in future studies.

In contrast to the use of DYD solely as LPS in AC-FET cycles, recent studies have demonstrated its effectiveness as a rescue strategy (28, 29). In line with findings from Labarta et al. (30), Mackens et al. (28) conducted a retrospective study showing that when low progesterone levels were detected on the day of FET, adding DYD resulted in pregnancy outcomes comparable to those with normal progesterone levels. Similar observations were reported in a more recent study by Metello et al. (29).

Additionally, combination strategies are gaining interest, particularly in settings where intensive progesterone monitoring is unavailable. A randomized controlled trial (RCT) by Vuong et al. (31) found that adding dydrogesterone to MVP in AC-FET cycles led to higher live birth rates (LBR) and reduced miscarriage rates. These findings were similarly reported in two retrospective studies by Lawrenz et al. (32) and Vidal et al. (33).

Congenital anomalies were comparable between the two groups. These findings are in line with the recent meta-analysis by Katalinic et al. (34) However, Henry et al. (35) reported a higher incidence of congenital malformations, mainly hypospadias and congenital heart defects, after dydrogesterone exposure in pregnancy compared with progesterone. These conflicting results necessitate cautious interpretation and underscore the need for further research to clarify potential risks.

Hypertensive disorders of pregnancy, including gestational hypertension, preeclampsia, and HELLP syndrome, appeared to be more prevalent in the MVP group. The high affinity of DYD for the progesterone receptor may lead to improved immunomodulatory adaptation, potentially resulting in better placental invasion and, consequently, a lower incidence of placenta-related disorders such as hypertensive disorders of pregnancy. A recent study by Melo et al. (18) demonstrated a protective effect of first-trimester exposure to progesterone on the development of hypertensive disorders of pregnancy. These observations require validation in larger studies powered to assess this specific outcome.

The effectiveness and overall satisfaction of both types of luteal phase support (LPS) were comparable. However, DYD demonstrated significantly greater convenience compared to MVP. This finding aligns with expectations and may be attributed to the preference for the oral route of LPS administration over the vaginal route.

The main limitation of this study is its exploratory design, with insufficient statistical power to draw definitive conclusions on the primary outcome, and the restriction to a single-center population. However the findings of this randomized study may serve as a base for further research in developing optimal dosing of different progesterone regimen in luteal phase support in artificially prepared FET cycles.

## 5 Conclusion

In conclusion, the findings of this RCT provide a foundation for further research into the optimal dosing regimen of DYD in artificial cycle frozen embryo transfer cycles, particularly given the greater convenience associated with oral LPS compared to vaginal administration. However, based on the current evidence, we suggest to be cautious using DYD at a dose of 10 mg three times daily as the sole LPS strategy in AC-FET cycles relying solely on exogenously administered progesterone, as clinically relevant differences in pregnancy loss and ongoing pregnancy rates were observed in this study. Despite this, DYD has shown utility as a rescue strategy in AC-FET cycles, as demonstrated in recent studies, and as a component of combination LPS protocols. Future investigations should focus on whether higher doses and more frequent administration of DYD can minimize plasma fluctuations and reduce pregnancy losses.

## Data Availability

The raw data supporting the conclusions of this article will be made available by the authors, without undue reservation.
