# Supplementary material for: Dydrogesterone versus micronized vaginal progesterone for luteal phase support in artificial cycle frozen embryo transfer (REMODEL): a pilot prospective randomized controlled trial
Source: Front Endocrinol (Lausanne). 2026 Apr 16;17:1785932. doi: 10.3389/fendo.2026.1785932 (PMC13130187; doi:10.3389/fendo.2026.1785932)
Supplement: Supplementary file 1 [file Table1.docx]

| Record ID | Treatment group | AE severity | AE description |
| --- | --- | --- | --- |
| 1 | B | Moderate | Hypertensive disorder of pregnancy (pre-eclampsia at 38weeks6days) |
| 6 | B | Moderate | Hypertensive disorder of pregnancy (Gestational hypertension) |
| 10 | A | Moderate | Migraine and vomiting |
| 13 | A | Moderate | Nausea and headache |
| 14 | A | Severe | Intra-uterine demise at 14 weeks’gestation and termination of pregnancy at 32w of second fetus because of schizencephaly |
| 34 | A | Moderate | Hypertensive disorder of pregnancy  (gestational hypertension) |
| 46 | A | Mild | Bladder infection |
| 53 | B | Mild | Blood loss first trimester |
| 60 | A | Moderate | Abdominal pain, fatigue, dizziness, headache, nausea, migraine |
| 62 | A | Mild | Blood loss first trimester |
| 66 | A | Moderate | Hyperemesis gravidarum |
| 71 | B | Mild | Dizziness |
| 73 | B | Mild | Nausea |
| 74 | B | Mild | Allergic reaction (vaginal itching, swelling, redness) |
| 77 | B | Mild | Dizziness |
| 92 | B | Mild | Nausea, diarrhea |
| 95 | B | Moderate | Migraine, swollen gums |
| 99 | A | Moderate | Fatigue, headache |
| 106 | A | Moderate | Lower abdominal pain, back pain |
| 111 | A | Mild | Migraine with aura |
| 128 | B | Mild | Headache, Nausea, Vaginal itching, Burning sensation |
| 132 | B | Moderate | Hypertensive disorder of pregnancy  (pre-eclampsia at 38weeks) |
| 133 | B | Moderate | Hypertensive disorder of pregnancy  (pre-eclampsia at 37weeks1day) |
| 137 | A | Severe | Ischemic cerebrovascular accident |
| 150 | B | Severe | Duodenal malrotation necessitation operation and hospitalization of the neonate |

Supplementary table 1: Overview of adverse events per treatment group

Group A: Dydrogesterone , Group B: Micronized vaginal progesterone
